# Trypanosomatid Richness in Wild and Synanthropic Small Mammals from a Biological Station in Rio de Janeiro, Brazil

**DOI:** 10.3390/pathogens10111442

**Published:** 2021-11-05

**Authors:** Alice Pereira Berbigier, Juliana Helena da Silva Barros, Edilene Sousa Pontes, Cristiane Varella Lisboa, Rosana Gentile, Samanta Cristina das Chagas Xavier, Ana Maria Jansen, André Luiz Rodrigues Roque

**Affiliations:** 1 Laboratório de Biologia de Tripanosomatídeos, Instituto Oswaldo Cruz, Fundação Oswaldo Cruz, Rio de Janeiro 21040-360, Brazil; aliceperber@gmail.com (A.P.B.); juliana.barros@ioc.fiocruz.br (J.H.d.S.B.); edilenespontes@gmail.com (E.S.P.); crisvarella@ioc.fiocruz.br (C.V.L.); samanta@ioc.fiocruz.br (S.C.d.C.X.); anamariajansen2@gmail.com (A.M.J.); 2 Laboratório de Biologia e Parasitologia de Mamíferos Silvestres Reservatórios, Instituto Oswaldo Cruz, Fundação Oswaldo Cruz, Rio de Janeiro 21040-360, Brazil; rgentile@ioc.fiocruz.br

**Keywords:** parasites, reservoirs, Atlantic Forest, anthropized areas, richness, taxonomy

## Abstract

Trypanosomatids are diverse and can infect several host species, including small mammals (rodents and marsupials). Between 2012 and 2014, 91 small mammals were surveyed for trypanosomatid infection in the Estação Biológica FIOCRUZ Mata Atlântica (EFMA), an Atlantic Forest area in Rio de Janeiro that presents different levels of conserved and degraded areas. Blood, skin, liver, and spleen samples were submitted to parasitological, serological, and molecular assays to detect the infection and determine the taxonomic status of their parasites. Sixty-eight individuals (74.7%; *n* = 91) were infected by trypanosomatids, including fourteen mixed infected by different trypanosomatid parasites. These hosts were infected by: *T. cruzi* DTU TcI (*n* = 12), *T. cruzi* DTU TcIV (*n* = 2), *T. janseni* (*n* = 15), *T. dionisii* (*n* = 1), and *T. rangeli* A (*n* = 1) detected in blood or tissue cultures, in addition to *T. cruzi* DTU TcI (*n* = 9) and *Leishmania* sp. (*n* = 1) only by the molecular diagnosis. Serological diagnosis was positive in 38 (71.6%) individuals for *T. cruzi*, the same amount for *Leishmania* spp., and 23 (43.3%) individuals were mixed infected. These data indicate a remarkable richness of trypanosomatid species/genotypes infecting small mammals, even in a disturbed area with low mammal species diversity—as is the case of the EFMA—reinforcing the generalist aspect of these parasites.

## 1. Introduction

The *Trypanosomatidae* family (Protozoa: Trypanosomatida) comprises parasites from plants, invertebrates, and vertebrate animals that, according to their life cycles, can be classified as monoxenic or heteroxenic [1,2]. At least twenty-four genera are recognized within this family, Refs. [3,4,5] with the genera *Trypanosoma* and *Leishmania* being the most studied because of their medical and veterinary importance [2]. For example, the more than twenty species of *Leishmania* described as responsible for different clinical forms of human leishmaniasis [6]; *Trypanosoma evansi* [7], which is the causative agent of an equine disease called “mal-de-cadeiras” or “surra”; and *Trypanosoma cruzi*, the causative agent of Chagas disease, a heterogeneous parasite that can be classified into seven discrete typing units (DTUs): TcI-TcVI and Tcbat [8,9]. More than twenty *Leishmania* species described as responsible for different clinical forms of human leishmaniasis [6]; *Trypanosoma evansi* [7], which is the causative agent of an equine disease called “mal-de-cadeiras” or “surra”; *Trypanosoma cruzi*, the causative agent of Chagas disease, a heterogeneous parasite that can be classified into seven discrete typing units (DTUs): TcI-TcVI and Tcbat [8,9]. 

Rodents and marsupials are some of the hosts that can be involved in the transmission cycle of several trypanosomatid species. They have an important role in the maintenance of these parasites in the wild environment, acting as hosts and, in some scenarios, as reservoirs [10]. Rodents are the most diverse of all mammalian groups worldwide, and in South America, the subfamily Sigmodontinae encompasses 56% of rodent species [11]. Reports of trypanosomatid infections in rodents are extensive and diverse [12,13], and probably related to the different types of environments in which they explore, such as forests, open fields, grasslands, and both rural and urban areas. Indeed, reports of infections by different *Leishmania* species, by distinct DTUs of *T. cruzi*, and by other *Trypanosoma* species have been described in several rodent species [10,12,14,15].

Marsupials are known to be some of the most ancient hosts of trypanosomatid parasites in the Americas. Apart from the *Leishmania* species and *T. cruzi*, they were recently described to be infected by other *Trypanosoma* species, such as *Trypanosoma dionisii*, *Trypanosoma cascavelli*, and *Trypanosoma lainsoni*, previously associated with other vertebrate hosts: respectively, bats, snakes, and rodents [16]. In addition, new *Trypanosoma* species and/or genotypes have also been described in these hosts, such as *Trypanosoma janseni* and *Trypanosoma* sp. DID, as was named this recently described taxonomic unit. This indicates that, although marsupials are the most commonly studied hosts, unknown parasites are still quite often described for this group [13,16,17]. Among marsupials, the common opossums of the genus *Didelphis* stand out as potential reservoirs for different *Trypanosoma* and *Leishmania* species, in addition to being considered bioaccumulators of *T. cruzi* DTU TcI, supporting their role as reservoirs [8].

Distinct parasitological, molecular, and serological assays are employed to diagnose trypanosomatid infection in their hosts [16,18]. Parasitological diagnoses are the only method that can indicate the presence of trypanosomatids in tissues, i.e., the potential of a host to be a source of infection for vectors. In addition, cultures are the only tool that allows the isolation and morphological description of these parasites [17]. Molecular assays are sensible and specific, especially when using conserved molecular targets that, once genomically sequenced, are able to identify parasite species and even subpopulations that do not grow in culture media [16,19,20]. Serological diagnoses are very sensitive, but have limited specificity and are dependent on the availability of positive and negative controls for reactions and conjugates specific to the investigated mammalian species [18,21]. The association of these different diagnostic methods is necessary to identify hosts and to define their putative role in the transmission of such parasites [21].

The Atlantic Forest is one of the most diverse Brazilian biomes, even though it is also the most degraded due to anthropic actions. Its territorial extension originally covered the entire Brazilian coast and, currently, only 11% to 16% of the original forests remain, most of them restricted to governmental protected areas [22,23]. One of these environmental conservation units is the Parque Estadual da Pedra Branca (PEPB: Pedra Branca State Park) located at the Pedra Branca Massif, which is the largest urban forest in the Americas encompassing an area of 12,491.72 hectares in the West Zone of the municipality of Rio de Janeiro [24]. For this reason, several initiatives were proposed, aiming to mitigate the effects of human occupation in this environment, such as the implementation of a biological station named Estação Biológica FIOCRUZ Mata Atlântica (EFMA: Fiocruz Atlantic Forest Biological Station). The EFMA is a part of the campus FIOCRUZ Mata Atlântica (CFMA—FIOCRUZ Atlantic Forest Campus), and is currently an environmentally protected area surrounded by low-income communities [25,26,27]. In this area, several scientific research projects have been developed, including the monitoring of fauna [26] and its parasites [17,28].

In EFMA, infections by trypanosomatids were described in different hosts, such as bats, dogs, marsupials, and humans [17,25,27,29]. Remarkably, two new *Trypanosoma* species were described in this area—*T. janseni* and *Trypanosoma caninum*, [17,29]—showing that this area, although relatively small, may still present unknown trypanosomatid diversity. In this study, we evaluated trypanosomatid infections in rodents and marsupials collected in areas from EFMA with different habitat characteristics according to the level of anthropic influence. Infections were detected, employing parasitological, molecular, and serological assays, and parasites were identified by DNA sequence analysis.

## 2. Results

### 2.1. Small Mammals and Their Sampling Areas

The species *Didelphis aurita* (Wied-Neuwied, 1826) widely prevailed in the study area (*n* = 70), followed by *Akodon cursor* (Winge, 1887) (*n* = 7), *Rattus rattus* (Linnaeus, 1758) (*n* = 7), *Marmosa paraguayana* (Tate, 1931) (*n* = 4), *Oligoryzomys nigripes* (Olfers, 1918) (*n* = 2), *Monodelphis americana* (Müller, 1776) (*n* = 1), and *Metachirus myosurus* (Temminck, 1824) (*n* = 1). The most captured species, *D. aurita*, was collected in all expeditions: 19 in July 2012, 11 in November 2012, 9 in April 2013, 15 in July 2013, 15 in November 2013, and 5 in April 2014, including the 4 recaptures. A significantly larger number of small mammals captured was observed in peridomicile area A1 (*n* = 51) than in the other areas; namely, transition area A2 (*n* = 32) and preserved forest area A3 (*n* = 11) (χ2 = 12.372, *p* = 1.2607E-05, df = 2).

### 2.2. Infection Rates of Trypanosomatids

Despite the differences observed in the number of collected individuals, we did not observe a significant difference in trypanosomatid prevalence among the different environments: A1 (36/50, 72%, confidence interval: 57.5–83.7), A2 (23/30, 76.7%, CI: 57.7–90.1), and A3 (11/9, 81.8%, CI: 48.2–97.7) (χ2 = 0.07819, *p* = 0.96166, df = 2) (Table 1). Seventy-five specimens of marsupials and sixteen specimens of rodents collected were analyzed for trypanosomatids, totaling ninety-one individuals. Considering all of the host species, the total trypanosomatid prevalence was 74.7% (CI: 64.5–83.3). Trypanosomatid prevalence was similar for marsupials (76%, CI: 64.7–85.1) and rodents (68.7%, CI: 41.3–88.9), without significant difference (χ2 = 0.054569, *p* = 0.8153, df = 1). No significant difference was observed in trypanosomatid prevalence between male (73.6%, CI: 59.7–84.7) and female (76.3%, CI: 59.8–88.5) hosts (χ2 = 0.01261, *p* = 0.91059, df = 1).

Seventy-four percent (74.3%; 52/70) of the captured *D. aurita* were infected by any trypanosomatid parasite, detected by at least one of the employed diagnostic tests (Table 1). Four of seven specimens of *A. cursor* were found to be infected by trypanosomatids. All specimens of *R. rattus*, *M. myosurus*, and *M. paraguayana* analyzed were infected, and none of the *O. nigripes* were infected with trypanosomatids (Table 1). *M. americana* was not surveyed for trypanosomatid infection because it was found dead in the trap. No significant difference was observed in trypanosomatid prevalence for *D. aurita* between males (70.4%, CI: 54.8–83.2) and females (80.8%, CI: 60.6–93.4) (χ2 = 0.13239, *p* = 0.71596, df = 1) or between young (75%, CI: 58.8–87.3) and adult (73.3%, CI: 54.1–87.7) individuals (χ2 = 0.0036831, *p* = 0.95161, df = 1).

### 2.3. Parasitological and Molecular Diagnosis 

Eighty-five individuals were tested by fresh blood examination; of these, four (4.7%), all *D. aurita*, were positive for the presence of flagellates, two from A1: LBCE 17667 and 18228, and two from A2: LBCE 17674 and 18209. Two of them were also positive in other diagnostic tests: LBCE 18228 and 18209 were positive in serology for both *T. cruzi* and *Leishmania* sp., while the latter was also positive in hemoculture that was characterized as *T. cruzi*, DTU TcIV.

Twenty-five individuals (27.8%; *n* = 90) were positive in hemocultures: twenty-three *D. aurita*, one *M. paraguayana*, and one *A. cursor*. Of these, ten hemocultures were cryopreserved, and fifteen were characterized as employing culture sediments. The following parasites were characterized using the 18S rDNA molecular target: *T. cruzi* DTU TcI (*n* = 12), *T. cruzi* DTU TcIV (*n* = 2), *T. janseni* (*n* = 9), *T. dionisii* (*n* = 1), and *T. rangeli* lineage A (*n* = 1) (Table 2).

Two hundred and five samples of tissue fragments of spleen (*n* = 61), liver (*n* = 61), and skin (*n* = 83) were examined by culture, and six of them (2.9%) were positive: five from spleen and one from liver. These tissues were derived from five individuals, because one individual of the species *D. aurita* was also positive in the liver, in addition to the spleen. All of these tissue samples were characterized as *T. janseni* using the 18S rDNA molecular target, and all of them were cryopreserved, except for one spleen sample that was characterized as employing the culture sediment (Table 2). 

Concerning *Leishmania* sp. infection, only one spleen fragment derived from *A. cursor* (LBCE 18231) was positive in kDNA-PCR, but *Leishmania* species identification was not achieved because HSP70 (234)-PCR was negative. 

The 18S molecular target was also tested directly on DNA extracted from host tissues, and eighteen of them were positive: nine spleen, six skin, and three liver samples. These positive tissues were derived from twelve individuals, six of whom presented at least two positive tissues, with the spleen always associated with another tissue: skin in *M. myosurus* (*n* = 1), *D. aurita* (*n* = 1), *M. paraguayana* (*n* = 2), *A. cursor* (*n* = 1), and liver in another individual from *M. paraguayana* (*n* = 1) (Table 2). 

From these eighteen samples, nine were successfully characterized at the species level, all as *T. cruzi* DTU TcI, two of them employing the 24S molecular target, and the other nine samples that could not be characterized at the species level were defined as infected by Trypanosomatidae (Table 2).

### 2.4. Phylogenetic Analysis of Trypanosomatids Characterized at the Species Level

**Figure 1:**
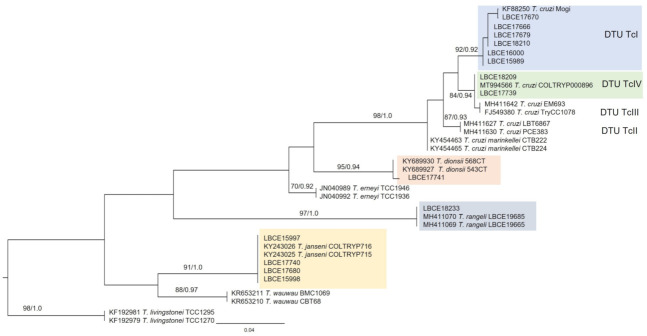
Phylogenetic analysis of 18S rDNA gene sequences by maximum likelihood (ML) and Bayesian (BI) inference analyses. The analysis indicates the phylogenetic position of trypanosomatids characterized as *T. cruzi* DTU TcI, *T. cruzi* DTU TcIV, *T. dionisii*, *T. rangeli*, and *T. janseni*. The maximum likelihood bootstrap values and Bayesian posterior probabilities are shown near the nodes. The numbers in the nodes indicate support per 5000 bootstrap in ML parsing. The scale bar shows the number of nucleotide substitutions per site. *Trypanosoma livingstonei* was used as an outgroup.

For the construction of the phylogenetic tree and analysis of the genetic distance between trypanosomatids characterized in this study, fourteen representative sequences of the thirty-nine samples sequenced at the species level were used: *T. cruzi* DTU TcI (6), *T. cruzi* DTU TcIV (2), *T. dionisii* (1), *T. rangeli* (1), and *T. janseni* (4) (Figure 1; Table 2). 

The reference sequences used in the phylogenetic tree construction come from the GenBank database and are presented with their respective accession numbers (Figure 1). These sequences were selected according to the percentage of identity and coverage among the generated gene sequences in this study with the gene sequences from GenBank.

### 2.5. Serological Diagnosis

Serological diagnosis was performed in 88 individuals, among which 53 (60.2%) were positive. Seropositivity was detected in 38 (71.6%, CI: 32.66–54.18) animals for both *T. cruzi* and *Leishmania* sp., of which 23 (43.3%, CI: 29.84–57.72) individuals had mixed infections (Table 3).

### 2.6. Recaptures

During the expeditions, four *D. aurita* individuals were recaptured, showing different results in the employed diagnostic assays:

1. LBCE 17825: captured twice in A2. It was positive in serology for *T. cruzi* and *Leishmania* spp., and *T. cruzi* DTU TcI was isolated from the blood. Four months later, only serological infection was observed, with the same IFAT titers (1/40 *T. cruzi*; 1/80 *Leishmania* spp.);
2. LBCE 17674: captured twice in A2. In the first capture, it was not positive for any of the diagnostic assays, and showed positivity in the fresh blood examination only in the second capture (nine months later);
3. LBCE 18232: captured twice in A1 (three months interval). It was positive in serology, for *T. cruzi* and *Leishmania* spp. The IFAT titers were different for *Leishmania* spp. (1:40 and 1:80), while the IFAT titers for *T. cruzi* remained the same (1:160);
4. LBCE 18255: first captured in A2, and four months later in A1 it was positive in serology for *T. cruzi*, showing different IFAT titers in the first (1:160) and second capture (1:80), while it was serologically positive for *Leishmania* spp. only in the first capture (1:40). In the second capture, *T. janseni* was isolated in the blood culture.

## 3. Discussion

*D. aurita* was the most abundant small mammal in the samplings from EFMA, probably because capture expeditions occurred during their reproductive period, described to begin in July/August and to finish in March [26]. As a consequence, most of the isolated parasites in this study were derived from this species, which certainly influenced the ecological pattern of the local enzooty. The sex and age of these hosts did not seem to significantly influence the prevalence of trypanosomatids. These individuals of *D. aurita* were found infected and may move among the three sampling areas, highlighting their potential for parasite dispersion [10,13].

In parasitological diagnoses, four individuals of *D. aurita* were considered positive in the fresh blood exam, and twenty-five individuals had trypanosomatids isolated by blood cultures: *D. aurita* (*n* = 23), *M. paraguayana* (*n* = 1), and *A. cursor* (*n* = 1). This indicates that they may be competent to be a source of infection for vectors and/or infect other mammals if predated [16,31]. *T. cruzi* DTU TcI prevailed (*n* = 12) and was detected exclusively in *D. aurita*. *T. cruzi* DTU TcIV, was detected in individuals of *D. aurita* and *M. paraguayana*, and had previously been reported in bat species in the EFMA [27], indicating a broad host distribution in this area. Both DTUs were previously described in the Atlantic Forest: *T. cruzi* TcI is the most ubiquitous genotype, and was found infecting distinct mammalian orders in several areas from the Brazilian Southeastern Atlantic Forest: São Paulo, Espírito Santo, and Rio de Janeiro states. The *T. cruzi* TcIV genotype is widely reported in other Brazilian biomes and was also detected in Atlantic Forest fragments from Espírito Santo and other locations in Rio de Janeiro state. Moreover, *T. cruzi* DTUs TcII and TcIII were also previously reported in Atlantic Forest areas [10,27,32,33,34].

*Trypanosoma janseni* was first described at EFMA in spleen and liver cultures from an individual of *D. aurita*: LBCE 17665 [17]. This parasite has also been detected in other studies in blood clots from *D. aurita* and in dogs [16,35]. In this study, this parasite was isolated in spleen cultures (*n* = 3) and, for the first time, in one hemoculture. These samples come from three individuals of *D. aurita*, where one of these individuals was infected both in the spleen and the blood. This parasite was also detected in the spleen (*n* = 1) and blood (*n* = 8) of nine other individuals of *D. aurita*, but these cultures were not established. Later, in expeditions conducted in June and November 2017, *T. janseni* was once more detected in the other four individuals captured close to A3, more precisely above 100 m height (data not shown). These data indicate that this parasite is established in all three sampling environments at EFMA, and that these hosts can be a source of *T. janseni* infection for its potential (and still unknown) vectors. 

*T. dionisii* is usually associated with bat species, and was previously reported in bats from EFMA [27]. Recently, this parasite has also been detected in other distinct groups of hosts, such as marsupials and even humans [16,32]. In this study, we detected *T. dionisii* in a non-bat species for the first time in EFMA; in this case, *D. aurita*. This reinforces the idea that this parasite is probably more generalist than previously recognized. 

*Trypanosoma rangeli* has genetic heterogeneity, and can be grouped into five genotypes (A, B, C, D, and E) [36,37]. In this study, infection by *T. rangeli* lineage A was detected in blood samples from only one *D. aurita*, and this can be explained by the low parasitemia that this parasite presents in parasitological diagnoses, as reported by Dario et al. (2021) [38]. This parasite can be found in several species of mammalian hosts, having a large geographical distribution that has been reported in several locations [38,39], including other areas of the Atlantic Forest [33,34,38,40]. Despite this, this is the first time that lineage A has been reported in the state of Rio de Janeiro, where only lineages D and E were previously reported [38].

In the molecular diagnosis directly in tissues, trypanosomatid infections were detected in eighteen tissue samples, with the spleen having the largest number of positive samples, followed by the liver and skin. Of these, nine were characterized as *T. cruzi* DTU TcI, and the other nine were maintained as Trypanosomatidae because it was not possible to characterize them at the species level. This is probably related to the quality of the amplified DNA and the presence of host DNA in the tissue samples, as these had poor and/or unspecific bands in the agarose gel, even after the two steps of DNA amplification by nested-PCR, hindering the purification and sequencing processes. When sequenced, these samples had electropherograms with very high and/or very low peaks, indicating that the DNA used in these reactions was not viable to generate good sequencing and, consequently, characterize the parasites present in these tissues at the species level. Positivity in 18S PCR added important information because these samples belonged to four individuals who were negative in other diagnostic assays, highlighting the efficiency of the molecular diagnosis in detecting trypanosomatid infections.

Molecular detection and parasite characterization from host tissue samples also allowed the detection of *T. cruzi* DTU TcI in other hosts in addition to *D. aurita*, such as *A. cursor* and *M. paraguayana*. DTU TcI was the most prevalent parasite subpopulation infecting small mammals at EFMA, and in fact, this DTU is the most common in the wild transmission cycle [8]. It is important to note that DTU TcI was detected mainly in individuals of *D. aurita* (and only isolated in this species). This may be related to the fact that the genus *Didelphis* can present great infectivity potential and high levels of parasitemia, especially when infected by *T. cruzi* belonging to this DTU [8,10]. Regarding the phylogenetic analysis of these characterized trypanosomatids, it is possible to observe through their phylogenetic positions that all these species and their related taxonomic units belong to the *Trypanosoma cruzi* clade.

Serological diagnosis (IFAT) presented the highest number of positive samples (*n* = 53) in comparison to the other diagnostic methods. According to Roque and Jansen (2014) [13], serology indicates host exposure to the parasite at some moment in its lifetime, but is not able to confirm the maintenance of the parasite in the host or its potential to act as a source of infection for vectors. Animals that had a positive diagnosis only by serological assays are considered to have low potential to transmit the parasite to vectors. 

The *A. cursor* spleen sample (LBCE 18231) that was positive in kDNA-PCR was negative in HSP70 (234)-PCR, probably because the kDNA molecular target has more copies (estimated 10–20 thousand mini-circle copies) of genome compared to the other molecular targets, such as the HSP70 molecular target (234), increasing chances of amplification [41,42,43].

Twenty-five individuals were positive in at least two of the diagnostic assays performed. Among them, fourteen were mixed infections with more than one different trypanosomatid species. These infections can modulate the infections detected in the serological diagnosis, where cross reactions can interfere with the titers of detected parasites, and in the parasitological diagnosis; for example, in fresh blood examination, where only parasites with high parasitemia will be able to be detected. This high rate of mixed infections is probably related to their ecological habits (diet, for example), because most of these small mammals are omnivorous and may prey on insects infected by these parasites. In addition, it is worth mentioning that there was a significant difference in prevalence rates considering the different diagnostic methods used, which reinforces the importance of combining the use of these diagnostic assays. In addition to improving the detection of different trypanosomatid species, the etiological agent was confirmed in some infections previously detected only by serology and fresh blood examination.

*Trypanosoma cruzi* is a paninfective parasite that is able to infect all nucleated mammalian cells in addition to blood. The most common tissue in which it is detected is blood, but other tissues, such as the liver and spleen, can also present parasites, probably in amastigote nests [10]. *Trypanosoma janseni* is less known, and was first described in spleen and liver fragments [17], but was isolated in blood for the first time in the present study. These parasites, as well as *T. rangeli* and *T. dionisii*, share the same mammalian host in the area (sometimes the same individual, as presented), but almost nothing is known concerning the consequences of the mixed infection in the course of infection or in the success to be transmitted. Understanding the influence of mixed infection on parasite fitness in natural conditions is a quite difficult aspect that parasitologists must face.

Among the four individuals of *D. aurita* that were recaptured, three were positive in the serological diagnosis for *T. cruzi* and *Leishmania* spp. in both captures, showing that the antibody levels were maintained over time (up to nine months). Three of them had positive results by the parasitological diagnoses:

1. positive in the fresh blood examination in the second capture, probably showing an increase in parasitemia or infection by other trypanosomatid parasites;
2. positive blood culture for *T. cruzi* DTU TcI only in the first capture, with the expected decrease in parasitemia in the late phase of infection, as this parasite was not detected in the second capture; and
3. positive blood culture for *T. janseni* only in the second capture, probably because that host became infected after the first capture. The latter was recaptured after four months in a different area. This result indicates that individuals of *D. aurita* can move across different areas in the study site. This is not a surprising finding considering that *D. aurita* commonly covers long distances during its lifetime [13,44].

This study showed that even in an area that has high levels of human disturbance and low richness of mammalian species, as is the case of EFMA, it was possible to detect a remarkable richness of trypanosomatid species, especially when using different diagnostic methods. In addition, some of the infected small mammals displayed infection patterns (detectable parasitemia) that highlighted their potential to act as reservoirs in space and time. *D. aurita*, which presented high levels of infection, moved across areas, potentially allowing parasite dispersion. This fact corroborates the nonsignificant difference observed in trypanosomatid prevalence among peridomicile, transition, and preserved forest environments. Furthermore, all rodent species captured are either synanthropic (*R. rattus*) or opportunistic (*A. cursor* and *O. nigripes*), the two latter occurring in several kinds of habitats, including rural and urban areas. The urban expansion that has been occurring in the surroundings of EFMA is also an important factor that directly affects small mammal richness and the transmission of their parasites, especially considering the dwellings and domestic animals present in the area, representing an interface region between urban and sylvatic environments. 

In this area of EFMA, *D. aurita* proved to be an important reservoir for *T. cruzi* and *T. janseni*, and presented detectable parasitemia for *T. dionisii* and *T. rangeli*, as demonstrated by positive hemocultures. These ancient trypanosomatid hosts can be found near human dwellings and serve as a source of infection for vectors in this area. It was already reported that these animals may present (and eliminate) metacyclic infective forms of *T. cruzi* in their scent glands, but this trait was not yet observed for other *Trypanosoma* species. The consumption of opossum meat by the local population has not been reported, which would also represent a potential risk for human infection due to the manipulation of infected blood. Except for *T. cruzi*, infection by the other *Trypanosoma* species observed in EFMA was not observed (*T. janseni*) or is not described as pathogenic for humans (*T. rangeli* and *T. dionisii*). Infection by *Leishmania* sp. was observed in only one rodent species, but it is worth mentioning that there are human and canine cases of Tegumentary Leishmaniasis in the area [25,26,27], demonstrating the risk of occurrence of mixed infections by distinct trypanosomatid parasites (as observed in the local fauna), also in humans. All these factors may favor the establishment of zoonotic transmission involving some of these trypanosomatids, and highlight the importance of long term monitoring of zoonosis in wild and synanthropic reservoirs in the region. 

## 4. Materials and Methods

### 4.1. Study Area

The study was conducted in EFMA (22°56′25″ S, 43°24′18″ W), which is partially located in PEPB, Rio de Janeiro, Brazil (22°56′23″ S, 43°24′12″ W). EFMA became a biological station administered by Fundação Oswaldo Cruz in 2016, being the first biological station in Rio de Janeiro and the first administered by the Brazilian Ministry of Health, representing a unique potential for research and interdisciplinary actions of environmental health, urban planning, and public policies [45,46].

**Figure 2:**
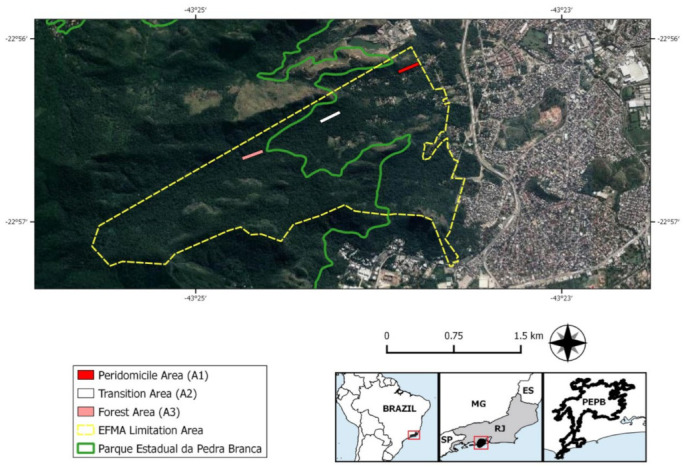
Map indicating the limits of FIOCRUZ Atlantic Forest Biological Station (EFMA) (yellow surrounded area), partially inserted in Pedra Branca State Park (PEPB) (green surrounded area). The three environments of small mammal captures are marked by colored lines: peridomicile (A1) in red, transition (A2) in white, and preserved forest (A3) in pink. Satellite images of the collection environments were obtained using Terra Incognita™ software, version 2.45. Vector layers of the municipal limit of the Rio de Janeiro state were obtained from Brazilian Institute of Geography and Statistics, the limit of PEPB was obtained from Ministry of Environment, and limits of EFMA areas were assigned by EFMA team.

EFMA comprises preserved areas with secondary vegetation of Atlantic Forest surrounded by degraded areas partially occupied by low-income communities. The study was performed in three environments, according to distinct levels of human disturbance (Figure 2):

1. peridomicile (A1): representing areas adjacent to human dwellings;
2. transition (A2): disturbed forest and reforestation areas between the peridomicile and the preserved forest; and
3. preserved forest (A3): the most preserved and distant area from the human dwellings composed of a secondary forest of ombrophilous dense vegetation. See Gentile et al. (2018) [26] for a better description of the study areas.

### 4.2. Small Wild Mammal Capture and Identification

Rodents and marsupials were captured using Sherman™ (Model XLK; 3 × 3.75 × 12 in.; Tallahassee, FL, USA) and Tomahawk™ (Model 201; 16 × 5 × 5 in.; Hazlehurst, WI, USA) live-traps placed on the floor and baited with a mixture of banana, bacon, peanut butter, and oats. Traps were settled in six linear transects, two in each area. Each transect had 20 capture points spaced 10 m apart. The captures occurred in six expeditions conducted in July and November 2012, April, July, and November 2013, and April 2014. The total capture effort was 1200 trap-nights/sampling section (totaling 7200 trap-nights during the six sampling sections). For every captured animal, the trap was identified, placed in a plastic bag, and taken to a field laboratory. 

The identification of small mammal species was carried out using external and cranial morphological characters, with the exception of the genus *Akodon* Meyen, 1833, whose species were identified by their diploid number after karyotyping [26]. Lactating females, young animals, and specimens that exceeded the limit of the capture license were tagged with earrings and released at their capture points. Some of these individuals were later recaptured. Euthanized small mammals were taxidermized and deposited as voucher specimens in the scientific collection (Coleção de Mamíferos do Museu Nacional) of the Department of Vertebrates at the National Museum of Rio de Janeiro (Museu Nacional, Universidade Federal do Rio de Janeiro) [26].

### 4.3. Field Procedures

Blood samples were obtained through cardiac puncture of the captured small mammals after anesthesia with ketamine hydrochloride (10–30 mg/kg), associated with xylazine (2 mg/kg) for marsupials (1:1) or associated with acepromazine (5–10 mg/kg) for rodents (9:1). The collected blood samples were employed for parasitological analysis (fresh blood examination and hemoculture) and serology. 

A drop of approximately 5 µL of blood was placed between the slide and coverslip for fresh blood examination. For hemoculture, approximately 0.6–0.8 mL of blood from each animal was divided into two tubes containing NNN (Nicolle, Novy, and McNeal) and LIT (Liver Infusion Triptose) culture medium [47]. For serology, blood was centrifuged (4000 G/5 min) to obtain serum, which was stored at −20 °C.

The captured small mammals previously anesthetized were euthanized with the intracardiac use of potassium chloride 19.1% for the collection of fragments of spleen, liver, and skin tissues [31]. Young and lactant *D. aurita* and individuals exceeding the limit of the capture license were examined, had blood samples collected, were marked by ear-tags, and were released at their trapping points. When possible (depending on the size of the animal), skin samples, with subsequent suturing, were collected.

The collected tissues were stored in tubes containing:

1. sterile saline (sodium chloride-NaCl at 58.44 g/mol), antibiotics, and antifungals (10 mg streptomycin, 25 µL amphotericin B, and 10,000 IU penicillin per mL, Sigma™, St, Louis, MO, USA commercial solution) for culture; and
2. absolute ethanol that was stored in a freezer at −20 °C for subsequent molecular diagnosis.

### 4.4. Parasitological Procedures

Fresh blood examination was performed in the field laboratory by the observation of a drop of blood on microscope slides using optical microscopy (400×). The samples that presented flagellates morphologically compatible with trypanosomatids were considered positive [48].

Hemocultures were observed every two weeks for up to five months [49]. The tissue samples were maintained in saline solution at 4 °C for 24 h and then transferred to culture tubes containing NNN medium and Schneider liquid medium [31]. The tissue cultures were observed twice a week for one month. 

Positive cultures were amplified and cryopreserved in the *Trypanosoma* Collection of Wild and Domestic Mammals and Vectors (Coleção de *Trypanosoma* de Mamíferos Silvestres, Domésticos e Vetores -ColTryp). The positive cultures that were not able to grow and amplify in culture medium were centrifuged to obtain the sediments.

### 4.5. Serological Diagnosis

The serum samples from rodents and marsupials were tested by IFAT (indirect immunofluorescent antibody test) to detect the presence of anti- *T. cruzi* IgG and anti- *Leishmania* sp. IgG [50] in twofold serial dilutions. Antigens were prepared using a mix of *L. braziliensis* (IOC/L566; MHOM/BR/1975/M2903) and *L. infantum* (IOC/L579; MHOM/BR/1974/PP75) or a mix of *T. cruzi* DTUs TcI (TcI - M000/BR/1974/F; [51]) and TcII (MHOM/BR/1950/Y; [51]) for the diagnosis of *Leishmania* spp. and *T. cruzi*, respectively. Rodents were tested using a commercial anti-rat IgG conjugated with fluorescein isothiocyanate (Sigma™, St. Louis, MO, USA). Marsupial sera were tested using an intermediate anti-*Didelphis* sp. IgG that was produced in rabbits and a commercial anti-rabbit IgG conjugated with fluorescein isothiocyanate (Sigma™, St. Louis, MO, USA) [52]. The adopted cutoff values were 1/40 for marsupials and 1/10 for rodents [53].

### 4.6. Molecular Diagnosis

For the molecular characterization of cultures, positive samples were incubated with proteinase K and sodium dodecyl sulfate (SDS) [31]. Genomic DNA from these samples was extracted using the standard phenol–chloroform method [54].

Spleen, skin, and liver fragments from small mammals were previously rehydrated (washing three times with Milli-Q water) to remove ethanol and subjected to DNA extraction using the commercial Wizard Genomic DNA Purification Kit (Promega, Madison, WI, USA) following the manufacturer’s instructions. At the end of the extraction, the DNA was suspended in 100 μL of DNA hydration solution (DNA Rehydration Solution) and stored in a freezer at −20 °C until use [12]. Experimentally infected and uninfected hamster tissues from previous studies were used as positive and negative controls, respectively.

Four molecular targets were used in tissue samples for the diagnosis of trypanosomatids:

1. kDNA target, used to detect *Leishmania* spp. Infections;
2. HSP70 (234) target, employed in *Leishmania* spp. kDNA-positive samples;
3. the 18S rDNA target for the detection of Trypanosomatidae, and for the characterization of all positive culture samples; and
4. the 24S rDNA target for 18S-positive samples in which characterization was not possible due to the low quality of the DNA sequences obtained.

For *Leishmania* spp. diagnosis, DNA amplification was carried out using the commercial PCR pureTaq Beads kit (Illustra PureTaq Ready-To-Go PCR Beads™, GE Healthcare, Chicago, IL, USA), with primers targeting the conserved region of *Leishmania* sp. kDNA minicircle, with 120 base pairs (bp): forward (5’-GGG(G/T)AGGGGCGTTCT(C/G)CGAA-3′) and reverse (5’-(C/G)(C/G)(C/G)G)(A/T)CTAT(A/T)TTACACCAACCCC-3′), as described by Degrave et al. (1994) [55]. The reactions were carried out in an Eppendorf Nexus™ Thermocycler (Eppendorf, Stevenage, England), under the following conditions: 94 °C for 5 min, 30 cycles of 94 °C for 1 min, 60 °C for 1 min, and 72 °C for 30 sec; followed by a final extension of 72 °C for 5 min. Electrophoresis was conducted in 8% polyacrylamide mini-gels and revealed by the Silver Stain Plus™ kit (Bio Rad, Hercules, CA, USA), following the manufacturer’s instructions. A molecular weight of 50 bp (DNA Step Ladder) (Promega, Madison, WI, USA) was used. The same tissues from experimentally infected and uninfected hamsters from previous studies were also used as positive and negative controls, respectively, in PCR and electrophoresis. 

The HSP70 (234pb) PCR was conducted with the following primers: forward (5’-GGACGAGATCGAGCGCATGGT-3′) and reverse (5’-TCCTTCGACGCCTCCTGGTTG-3′) according to Da Graça et al. (2012) [56] with some modifications. These modifications included a concentration of MgCl_2_ of 2.5 mM instead of 2 mM, the annealing temperature in the cycling reduced from 59 °C to 58 °C for 1 min, and 10 min of final extension instead of 5 min. The samples were amplified in a Veriti™ thermocycler (Applied Biosystems, Waltham, MA, USA). After electrophoresis, the 8% polyacrylamide minigels were also stained with silver nitrate using a specific kit (DNA Silver Staining, GE Healthcare, Chicago, IL, USA). The molecular weight used was also the 50 bp marker (DNA Step Ladder) (Promega, Madison, WI, USA). The positive DNA extraction controls were used as a positive control of the reaction, and the reaction mix was used as a negative control.

Nested-PCR using the molecular target for a partial region of 18S rDNA (approximately 600 bp) consisted of two rounds, and was conducted according to Smith et al. (2008) [30] with the following changes:

1. a final volume of 25 µL was used, containing 13.5 µL of ultrapure water, 8.5 µL of Go Taq Master Mix (Promega, Madison, WI, USA), and 2 µL of DNA in both rounds;
2. in the first round, 0.5 µL of the primers TRY R and F (16 pmol) (Eurofins Genomics™, Val Fleuri, Luxembourg, Luxembourg) were used, and in the second round, 0.5 µL of the primers SSU R and F (16 pmol) (Eurofins Genomics™, Val Fleuri, Luxembourg, Luxembourg) were used; and
3. in the cycling condition, the initial denaturation occurred at 95 °C for 15 min, in a Swift™ Max Pro Thermal Cycler 16 thermal cycler (model SWT-MXP-BLC-1).

Positive products derived from 18S PCR were purified with an Illustra™ GFX™ PCR DNA/Gel Band Purification kit (GE Healthcare, Chicago, IL, USA) or Wizard SV Gel kit and Clean-up System PCR kit (Promega, Madison, WI, USA) following the manufacturer’s instructions. The products were then sequenced with the BigDyeTM Terminator v3.1 Cycle Sequencing Ready Reaction Kit (Applied Biosystems, Waltham, MA, USA) in an ABI3730 DNA Analyzer Automatic Sequencer (Applied) by the PDTIS/FIOCRUZ Sequencing Platform.

Tissue samples that were positive by 18S nested-PCR but presented very weak bands and/or many unspecific bands were subjected to new DNA extraction using the Qiagen—Dneasy™, (Hilden, North Rhine-Westphalia, Germany) Blood and Tissue Kit following the manufacturer’s instructions. The obtained DNA was then used in novel Nested-PCRs with the following modifications aiming to improve the quality of the amplified material: ≥90.00 ng/µL was applied to 2 µL of DNA/< 90.00 ng/µL, and was applied 5 µL of DNA, 8.5 µL, or 5.5 µL of ultrapure water (depending on the volume of the DNA sample used in the reaction), 12.5 µL of Go Taq Master Mix, and 1.0 µL of primers R and F (TRY/SSU) at 20 pmol, with the following cycling condition: initial denaturation at 94 °C for 3 min, followed by 35 cycles at 94 °C for 30 s, 55 °C for 60 s, and 72 °C for 90 s. The final extension was at 72 °C for 10 min. From these samples, three could be characterized at the species level. 

The PCRs targeting the 24S rRNA, which corresponds to the large ribosomal subunit and can amplify from 200 to 300 bp, were used to detect trypanosomatid infections in the nine samples that could not be characterized at the species level by the 18S molecular target. The reactions occurred following Arruda et al. (1990) [57] with modifications: 5 µL of DNA, 12.5 µL of GoTaq Master Mix, and 2 µL of primers (Invitrogen™, Waltham, MA, USA) F D75 (5′-GCAGATCTTGGTTGGCGTAG-3′) and R D76 (5′-GGTTCTCTGTTGCCCCTTTT-3′) at 10 pmol and 3.5 µL of ultrapure water. The cycling conditions were as follows: 1 cycle at 94 °C for 15 min, 3 cycles at 94 °C for 1 min, 60 °C for 1 min, 72 °C for 1 min; 3 cycles at 94 °C for 1 min, 58 °C for 1 min, 72 °C for 1 min; 3 cycles at 94 °C for 1 min, 56 °C for 1 min, 72 °C for 1 min; 3 cycles at 94 °C for 1 min, 54 °C for 1 min, 72 °C for 1 min; 35 cycles at 94 °C for 1 min, 52 °C for 1 min, 72 °C for 1 min; 1 cycle at 72 °C for 10 min. 

The purification and sequencing of the PCR products from the 24S rRNA molecular target were performed as described for the 18S molecular target. For 18S and 24S molecular targets, the following reaction controls were used: *T. cruzi* DNA (Y strain) was used as a positive control, and ultrapure water was used as a negative control. Electrophoresis for 18S and 24S molecular targets occurred as follows: the amplified products were applied (5 μL) in a 2% TBE (Tris-Borato-EDTA) agarose gel and stained with GelRed Biotium. The gels were visualized on the Gel Logic 212 Pro photo documenter using the Carestream MISE program, using a molecular weight marker of 100 base pairs (bp) as a reference (Ludwig Biotecnologia, Alvorada, Rio Grande do Sul, Brazil).

### 4.7. Phylogenetic Analyses

The obtained consensus sequences were manually edited using the SeqMan-DNA Star Program [58], and compared for similarity with sequences deposited in the GenBank database from the National Center for Biotechnology Information (NCBI) using the BLAST algorithm (Basic Local Alignment Search Tool). For species identification, the following values were adopted: cover (≥97%), identity (≥97%), and E-value (0.0).

Phylogenetic analyses were performed using maximum likelihood (ML) and Bayesian inference (BI) to confirm the characterization of the trypanosomatid species from the 18S rDNA gene and assess their phylogenetic positions. The BI analysis occurred in MrBayes (Version 3.1.1) [59], which is included in TOPALi v.2.5 software. Two runs were performed with 1,000,000 generations, a sample frequency of 10 and a burning of 25%, using the model Hasegawa Kishino Yano + gamma distribution (HKY + G) with rate variation between sites.

The ML analysis was performed using the jModelTest v.2 program, which indicated the SYM+G4 model (Symmetrical Model plus 4 gamma distributed sites) using the Akaike information criterion (AICc Score) [60]. The construction of the ML tree was performed in the IQ-Tree [61,62] which is available in PhyloSuite software. To branch support, ultrafast bootstrapping [63] of 5000 replications with 1000 maximum interactions and a minimum interaction coefficient of 0.99 was performed. To visualize the ML tree and its bootstrap values, FigTree software was used. The genetic distance was analyzed in the Mega X program, and representative sequences were used for the construction of the phylogenetic tree, among all the sequences obtained in this study.

### 4.8. Statistical Analysis

Marsupials and rodents that were positive in any of the performed diagnostic assays (parasitological, serological, and/or molecular) were considered infected by trypanosomatids. Characterizations at the species level and/or subpopulation in the infected tissues were obtained by DNA sequence analysis.

Prevalence rates of trypanosomatids were calculated as the proportion of the number of infected animals in relation to the total number of animals analyzed according to Bush et al. (1997) [64] for each host species and considering all of the hosts analyzed. For marsupial *D. aurita*, trypanosomatid prevalence was investigated in relation to host sex and age. Chi-squared contingency tests were performed to evaluate differences in the number of animals captured, and in trypanosomatid prevalence among areas, between rodents and marsupials, male and female hosts, and young and adult hosts. These analyses were carried out using Past software version 3.09 [65] considering a significance level of 5%.

### 4.9. Ethics Statement

Small mammals were captured in accordance with the Normative Instruction of the Brazilian Institute of the Environment and Renewable Natural Resources (IBAMA nº154/2007 Licenses 13373-1 e 19037-1), Chico Mendes Institute for Biodiversity and Conservation (ICMBIO, license number 13373), and Environmental Institute of Rio de Janeiro state (INEA, license number 020/2011). Procedures with animals were previously approved by the Animal Use Ethics Committee of the FIOCRUZ (CEUA/FIOCRUZ), license LW81-12.

## Figures and Tables

**Table 1:** Rodents and marsupials captured in three environments (peridomicile—A1, transition—A2, and preserved forest—A3) at EFMA, Rio de Janeiro (RJ), Brazil, between 2012 and 2014, and their infection rates by trypanosomatids.

Order (n)	Species	Infected/Total	A1	A2	A3
	*Akodon cursor*	4/7 (57.5%, CI: 18.4–90.1)	4/7 (57.5%)	-	-
Rodentia (16)	*Oligoryzomys nigripes*	0/2 (0%, CI: 00.0–84.2)	0/1 (0%)	0/1 (0%)	-
	*Rattus rattus*	7/7 (100%, CI: 100–100)	5/5 (100%)	2/2 (100%)	-
	*Didelphis aurita*	52/70 (74.3%, CI: 62.4–83.9)	26/36 (72.2%)	20/26 (76.9%)	6/8 (75%)
Didelphimorphia (75)	*Marmosa paraguayana*	4/4 (100%, CI: 100–100)	1/1 (100%)	-	3/3 (100%)
	*Metachirus myosurus*	1/1 (100%)	-	1/1 (100%)	-
91	6	68/91 (74.7%)	36/50 (72%)	23/30 (76.7%)	9/11 (81.2%)

**Table 2:** Small mammal (rodents and marsupials) infection by Trypanosomatids in parasitological and molecular assays at EFMA, Rio de Janeiro (RJ), Brazil, between 2012 and 2014.

Collection Environment	Sample ID	Species	Molecular Analysis of the Trypanosomatids Detected in Cultures	Trypanosomatids Detected in Molecular Diagnosis Directly in Tissues	GenBank Access Number	COLTRYP Number
A1	LBCE 15991	*Akodon cursor*	Negative	Trypanosomatidae (Sp)/*T. cruzi* DTU TcI **** (S)	MZ221936 (S)	-
	LBCE 15994	*Akodon cursor*	Negative	*T. cruzi* DTU TcI *** (Sp)	MZ229972 (Sp)	-
	LBCE 15995	*Rattus rattus*	Negative	*T. cruzi* DTU TcI *** (L)	MZ229973 (L)	-
	LBCE 15997	*Didelphis aurita*	*T. janseni* (B) *	Negative	MZ541906 (B)	-
	LBCE 15998	*Didelphis aurita*	*T. janseni* (B) *	Negative	MZ541913 (B)	-
	LBCE 17670	*Didelphis aurita*	Negative	*T. cruzi* DTU TcI **** (L)	MZ221942 (L)	-
	LBCE 17677	*Didelphis aurita*	*T. cruzi* DTU TcI (B)	Negative	MZ541920 (B)	C00495
	LBCE 17680	*Didelphis aurita*	*T. janseni* (B, Sp)	Negative	MZ541926 (B)/MZ541914 (Sp)	C00494 (B)
	LBCE 17683	*Didelphis aurita*	*T. cruzi* DTU TcI (B)	Negative	MZ541921 (B)	C00501
	LBCE 17733	*Didelphis aurita*	*T. janseni* (B) *	Negative	MZ541910 (B)	-
	LBCE 17735	*Didelphis aurita*	*T. cruzi* DTU TcI (B)	Negative	MZ541900 (B)	C00538
	LBCE 17736	*Akodon cursor*	*T. janseni* (B) *	Negative	MZ541911 (B)	-
	LBCE 17737	*Didelphis aurita*	*T. janseni* (B) *	Trypanosomatidae (Sp)	MZ541909 (B)	-
	LBCE 17739	*Marmosa paraguayana*	*T. cruzi* DTU TcIV (B)	Negative	MZ541899 (B)	C00561
	LBCE 17742	*Didelphis aurita*	*T. cruzi* DTU TcI (B)	Negative	MZ541923 (B)	C00564
	LBCE 18203	*Didelphis aurita*	*T. cruzi* DTU TcI (B)	Negative	MZ541922 (B)	C00521
	LBCE 18233	*Didelphis aurita*	*T. rangeli* A (B) *	Negative	MZ541903 (B)	-
	LBCE 18241	*Didelphis aurita*	*T. janseni* (B) *	Negative	MZ541925 (B)	-
	LBCE 18243	*Didelphis aurita*	*T. janseni* (Sp)	Negative	MZ541916 (B)	C00911
	LBCE 18245	*Didelphis aurita*	*T. janseni* (B) *	Negative	MZ541912 (B)	-
A2	LBCE 15989	*Didelphis aurita*	*T. cruzi* DTU TcI (B) *	Negative	MZ541904 (B)	-
	LBCE 15990	*Didelphis aurita*	*T. cruzi* DTU TcI (B) *	Negative	MZ541905 (B)	-
	LBCE 15999	*Metachirus myosurus*	Negative	Trypanosomatidae (Sp, S)	-	-
	LBCE 16000	*Didelphis aurita*	*T. cruzi* DTU TcI (B) *	*T. cruzi* DTU TcI **** (Sp, S)	MZ541907(B)/MZ221937 (Sp)/MZ221941 (S)	-
	LBCE 17665	*Didelphis aurita*	*T. janseni* (Sp, L) **	Negative	KY243025 (Sp)/KY243026 (L)	-
	LBCE 17666	*Didelphis aurita*	Negative	*T. cruzi* DTU TcI (Sp)	MZ221938 (Sp)	-
	LBCE 17675	*Didelphis aurita*	Negative	Trypanosomatidae (S)	-	-
	LBCE 17729	*Didelphis aurita*	*T. cruzi* DTU TcI (B) *	Negative	-^2^	-
	LBCE 17740	*Didelphis aurita*	*T. janseni* (Sp)	Negative	MZ541915 (B)	-
	LBCE 17741	*Didelphis aurita*	*T. dionisii* (B) *	Negative	MZ541908 (B)	-
	LBCE 17823	*Didelphis aurita*	*T. cruzi* DTU TcI (B)	Negative	MZ541919 (B)	C00493
	LBCE 18209 ^1^	*Didelphis aurita*	*T. cruzi* DTU TcIV (B) *	Negative	MZ541901 (B)	-
	LBCE 18210	*Didelphis aurita*	*T. cruzi* DTU TcI (B)	Negative	MZ541902 (B)	C00520
	LBCE 18251	*Didelphis aurita*	*T. janseni* (Sp) *	Negative	MZ541917 (Sp)	-
	LBCE 18255	*Didelphis aurita*	*T. janseni* (B) *	Negative	MZ541918 (B)	-
A3	LBCE 17678	*Marmosa paraguayana*	Negative	Trypanosomatidae (Sp,S)	-	-
	LBCE 17679	*Marmosa paraguayana*	Negative	*T. cruzi* DTU TcI (Sp)/Trypanosomatidae (S)	MZ221939 (Sp)	-
	LBCE 17685	*Marmosa paraguayana*	Negative	*T. cruzi* DTU TcI (Sp)/Trypanosomatidae (L)	MZ221940 (Sp)	-
	LBCE 17743	*Didelphis aurita*	*T. cruzi* DTU TcI (B)	Negative	MZ541924 (B)	C00565
A1 = 20 A2 = 15 A3 = 4	*N* = 39	5 species	*T. cruzi* DTU TcI (12) *T. cruzi* DTU TcIV (2) *T. janseni* (15) *T. dionisii* (1) *T. rangeli* A (1)	Trypanosomatidae (9) *T. cruzi* DTU TcI (9)	Sequences (39)	Isolates (11)

A1, peridomicile area; A2, transition area; A3, forest area; (B) Blood; (Sp) Spleen; (S) skin; (L) liver. ^1^ Positive sample also in the fresh blood examination. ^2^ The sequencing of this sample was not deposited in the GenBank database, as the sequence was not ideal to be deposited, even after repeating the sequencing process. * Sediments. ** Samples that have previously been published by Lopes et al., 2018. *** Samples characterized by molecular target 24S. **** Samples characterized by changes in the PCR protocol (target 18S). All others were characterized by the molecular target 18S without any change in their original protocol [30].

**Table 3:** *T. cruzi* and *Leishmania* spp. infection in small mammals detected by indirect immunofluorescent assay test (IFAT) at EFMA, Rio de Janeiro (RJ), Brazil, between 2012 and 2014.

Infected Species (*n*; %)	*T.cruzi* (*n*; %) IFAT Titer Range	*Leishmania* spp. (*n*; %) IFAT Titer Range	Mixed Infection * *n* (%)
*Akodon cursor* (1; 14.3%)	(1; 100%) 1/10	(1; 100%) 1/20	1 (100%)
*Rattus rattus* (7; 100%)	(5; 71.4%) 1/10–1/40	(5; 71.4%) 1/10–1/20	3 (42.8%)
*Didelphis aurita* (42; 60%)	(29; 69.4%) 1/40–1/160	(31; 73.8%) 1/40–1/160	18 (42.8%)
*Marmosa paraguayana* (3; 75%)	(3; 100%) 1/40–1/160	(1; 33.3%) 1/80	1 (33.3%)
53/88 (60.2%)	38/53 (71.6%)	38/53 (71.6%)	23/53 (43.3%)

* Individuals also included in the counts of infections by *T. cruzi* and *Leishmania* spp.

## Data Availability

The sequences that were generated in this study are openly available on GenBank database (https://www.ncbi.nlm.nih.gov/genbank/) (accessed on 15 October 2021). Access numbers for 18S rDNA and 24S rDNA are provided in Table 2.
